# The development of one-day surgical care in Hungary between 2010 and 2019

**DOI:** 10.1186/s12913-022-08102-2

**Published:** 2022-06-20

**Authors:** Róbert Pónusz, Dóra Endrei, Dalma Kovács, Evelin Pónusz, Bence Kis Kelemen, Diána Elmer, Noémi Németh, András Vereczkei, Imre Boncz

**Affiliations:** 1 Institute for Health Insurance, Faculty of Health Sciences, University of Pécs, Vörösmarty street 3, Pécs, 7621 Hungary; 2 Real World & Big Data Health-Economics Research Centre, Faculty of Health Sciences, University of Pécs, Vörösmarty street 3, Pécs, 7621 Hungary; 3 National Laboratory for Human Reproduction, University of Pécs, Ifjúság street 20, Pécs, 7624 Hungary; 4 Department of International and European Law, Faculty of Law, University of Pécs, 48 square 1, Pécs, 7622 Hungary; 5 Department of Surgery, Clinical Centre, Medical School, University of Pécs, Ifjúság street 13, Pécs, 7624 Hungary

**Keywords:** Health policy reform, Diagnosis-related groups, Financing, Case mix index, Hungary

## Abstract

**Background:**

The constant increase in the utilization of one-day surgical care could be identified since more than a decade in most of European countries. Initially, according to the international rankings, the exploitation of one-day surgery in Hungary was not really significant. In 2010, the Hungarian policy makers intended to increase one-day surgical care as a priority strategy. The aim of our study was to analyze the evolution of the Hungarian one-day surgical care during the last decade in DRG- based performance financing system in Hungary.

**Methods:**

The dataset of the research was provided by the National Health Insurance Fund Administration of Hungary. The most important indicators related to the one-day surgical care were compared to inpatient care (market share, number of cases, and DRG cost-weights). To discover the impact of one-day surgical care to the utilization of inpatient treatment, the number of hospitalized days was also analyzed.

**Results:**

Between 2010 and 2019, the market share of one-day surgical cases increased from 42, to 80%. Simultaneously the constant increase of one-day surgical cases, the number of hospitalized days were decreased in inpatient care by 17%. The value of Case Mix Index has also increased, approximately by 140%, which could confirm that more complex interventions are being conducted in one-day surgical care as well.

**Conclusions:**

Due to the comprehensive health policy strategy related to the dissemination of one-day surgical care in Hungary, several important performance indicators were improved between 2010 and 2019. Given that Hungary belongs to the low- and middle-income countries, the results of the study could be considerable even in an international comparison.

## Background

Although one-day surgical care already appeared in the international academic literature at the beginning of the 1900s, in the early years it was considered to be safely applicable only for the simplest procedures and for patients in a very good overall condition [1, 2]. A recent guideline has indicated that one-day surgical treatment could be applicable for patient with stable medical conditions even if they have chronic disease [3]. In addition, studies highlighted the importance of pre-operative and pre-anesthetic assessment of the patients before the interventions in order to provide high patient safety and efficient treatment [4, 5].

There are several definitions for one-day surgical care. World Health Organization’s (WHO) definition is the following: “Day surgery or ambulatory surgery refers to the practice of admitting into hospital on the day of surgery carefully-selected and prepared patients for a planned, non-emergency surgical procedure and their discharge within hours of that surgery.” Procedures which were previously performed as inpatient cases are now considered appropriate for day surgery, while minor outpatient procedures and most day-case endoscopic procedures, which would never have involved admission, are excluded.” [ 6] International Association for Ambulatory Surgery (IAAS) defines either the fundamentals of the treatment and the exclusion criteria as well. According to the IAAS, “ambulatory surgery is an operation, excluding an office or outpatient procedure, where the patient is discharged on the same working day.” [7] It is important to highlight that there are several different denominations in the international medical and academic literature related to the one-day surgical treatment: ambulatory surgery, same-day surgery, day only, as well as day-case surgery [ 3, 7]. During this study the one-day surgical care terminology was applied.

The benefits of one-day surgical care compared to inpatient care have been discussed by numerous publications including the prevention of nosocomial infections [8], reduced accommodation, catering and laundry costs [9], high levels of patient safety [10], and shorter hospital stay for patients [11]. The above may also contribute to an increased level of trust among health care providers, policy makers, as well as patients which has undoubtedly facilitated the rapid spread of one-day surgical care. A prerequisite for the advancement of treatment modalities, however, is the development of surgical and anesthetic techniques, as has recently been emphasized in the international literature [12]. Although one of the main goals and benefits of one-day surgical care is the reduction of high inpatient care costs [13], one-day surgery remained to play a marginal role in recent decades, in higher-income countries as well [14]. The expansion of available information about one-day surgical care may have contributed to the significant increase in its proportion of inpatient care services performed within elective interventions during the 2000s [15]. A previously published paper highlighted that adequate funding techniques are essential for increasing the market share of one-day surgeries within elective interventions, as without suitable financing health care institutions will only have limited interest in performing one-day surgical treatment [16].

Diagnosis-Related Group- based (DRG) funding is widespread in Europe, and Hungary was one of the first countries where DRG-based funding was introduced into clinical practice and one-day surgical care as well [17–19].

DRG funding is a prospective payment method; its principle to refer the complexity and the cost requirements of the treatment related to the hospital cases. Similar cases form a homogeneous group which depends on the diagnosis of the patient, the related medical interventions, as well as the further status of the patient. For each DRG group has a cost-weight value which is previously defined by the national law [20]. One-day surgery was become one of the most supported health care treatments worldwide in recent years, and whereas the Hungarian performances was significantly behind the European benchmarks in this regard. Since the last decade several incentives were introduced, which are mainly linked to DRG-based financing techniques. Given the one-day surgical care and the inpatient treatment have common features in financial viewpoints, which based on the DRG financing system, the purpose of the study was to analyze the evolution of one-day surgical care through the past decade in the DRG- based financing system of Hungary.

### Characteristics of the Hungarian one-day surgical care and its financing

The Hungarian health care system is a solidarity-based national health insurance system with compulsory participation for every citizen. There is one purchaser, the NHIFA [21].

In Hungary, legislation defines the scope of one-day surgical care. Annex No. 9 to the 9/1993 (IV.2.) Public Welfare Ministerial Decree sets out the types of surgical procedures that could be performed in the form of a day surgery which could be eligible for public funding [22].

**Fig. 1:**
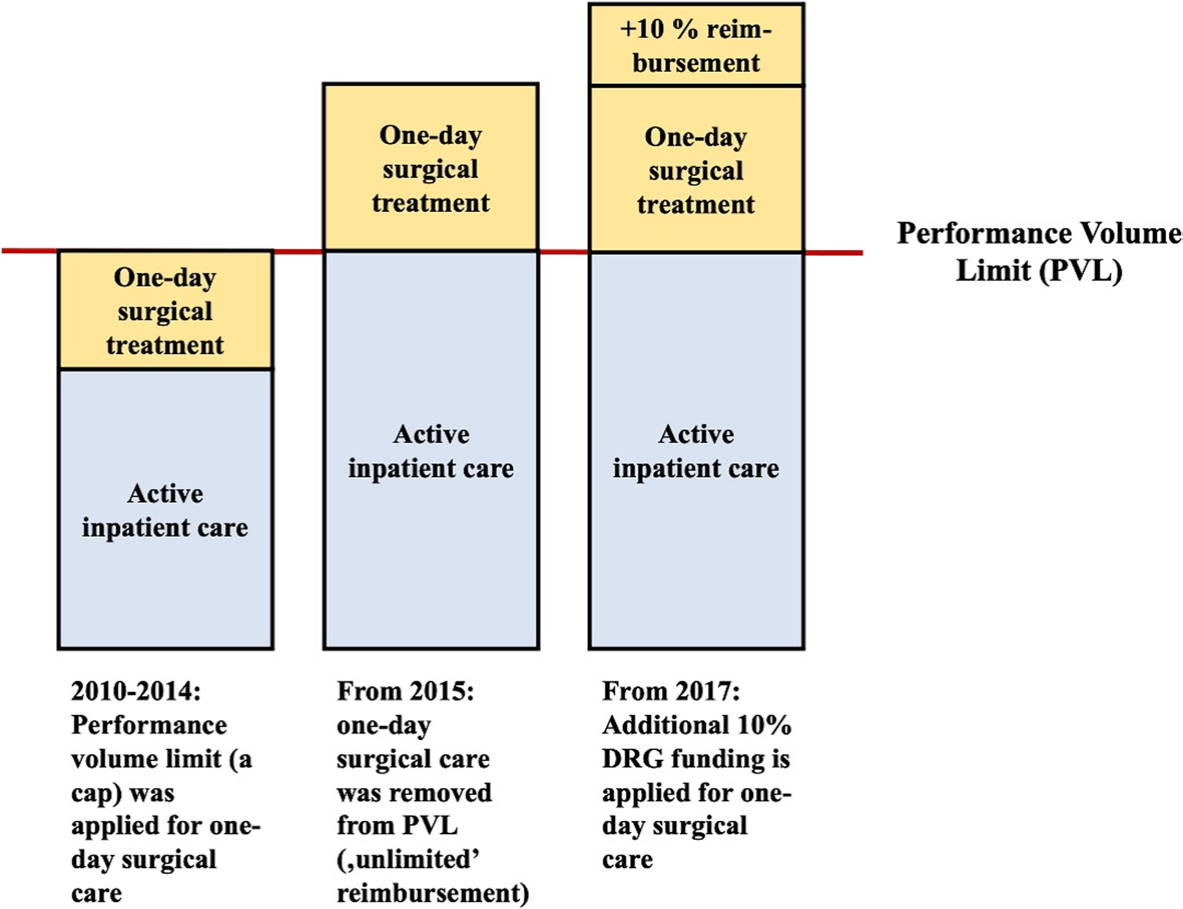
Introduction of the funding of Hungarian one-day surgical treatment

In 2010 there was 408 medical interventions which are eligible to deliver in one-day surgical treatment, on the other hand in 2019 the number of procedures was increased up to 781 interventions. In order to capture the most significant characteristics of the Hungarian one-day surgery and its treatment, it is essential to introduce those instruments which have the most fundamental influence on it. In the Hungarian public-funded inpatient care there is a Performance Volume Limit (PVL) which establishes the maximum performance of the hospitals individually. In Hungary between 2010 and 2019 there were 3 different policy decision with the purpose to enhance the market share of one-day surgical treatment. Firstly, from 2010 to 2014 there were an annual capacity redeployment from the unutilized inpatient DRG cost-weights to the proliferation of one-day surgical treatment. Secondly, in 2015, the previously applied PVL, a cap in one-day surgical treatment was removed, consequently, there was no limit how many one-day surgical procedures will be performed by the hospitals. Thirdly, the most recent, major policy measure became operative on 1 January 2017; an additional 10% funding DRG fee was introduced to further increase the number of one-day surgical interventions, if cases are delivered in one-day surgical treatment rather than inpatient care by Hungarian hospitals (Fig. 1).

Due to the change in financing technique, treatment on a day surgery basis has become more beneficial from a financial aspect for Hungarian hospitals, compared to inpatient care.

## Methods

We performed retrospective database study. Our database was provided by the National Health Insurance Fund Administration (NHIFA), the only health care financing agency in Hungary. The database included all case numbers, DRG cost-weights, number of hospital days from both one-day surgical and inpatient care between 2010 and 2019. Additionally, the research database also contained specifically defined cases and DRG cost-weights that were eligible to be financed as one-day surgical care. Furthermore, we also considered the gender and age of patients, the type of health care institution, the interventions according to International Classification of Procedures in Medicine (ICPM) performed as day surgery as well as the type of medical professional that carried out the intervention.

In the study, the market share of one-day surgical care relative to DRGs of surgical inpatient care by case and DRG cost-weight was evaluated. First, we divided the number of reported cases and DRG cost-weights of one-day surgery compared to the total number of surgical cases and DRG cost-weights of inpatient care. In addition, the annual ratio of one-day surgical cases and DRG-cost-weights were compared to the theoretical maximum of specified cases and DRG cost-weights according to the interventions listed in the Annex No. 9. of Public Welfare Ministerial Decree 9/1993 (IV.2.).

Taking into consideration the constant increase of the market share of one-day surgery cases, the number of hospital days, as well as the number of hospital days per patient were analyzed in inpatient care as well.

With respect to the research period, the Case Mix Index (CMI) value for one-day surgical care was annually determined. The CMI represents the average DRG-cost-weight per case, and it reflects the position of hospitals according to the progressivity level of health care institutions: the value of CMI is generally the lowest in small urban hospitals and the highest in clinical centers belonging to universities.

Subsequently, the mean age of patients in one-day surgical care by gender was defined. Our prior hypothesis was that with time, one-day surgery cases would shift towards including an older age spectrum, based on the continuous development of surgical and anesthetic techniques. We expected that the development in the above areas would also manifest in the fact that more complex interventions could be treated in the form of one-day surgeries. To determine which type of medical profession performed the most day surgeries, the market share of DRG cost-weights according to different medical fields was calculated.

The data were analyzed by Microsoft Excel and descriptive statistical tests were performed.

## Results

**Fig. 2:**
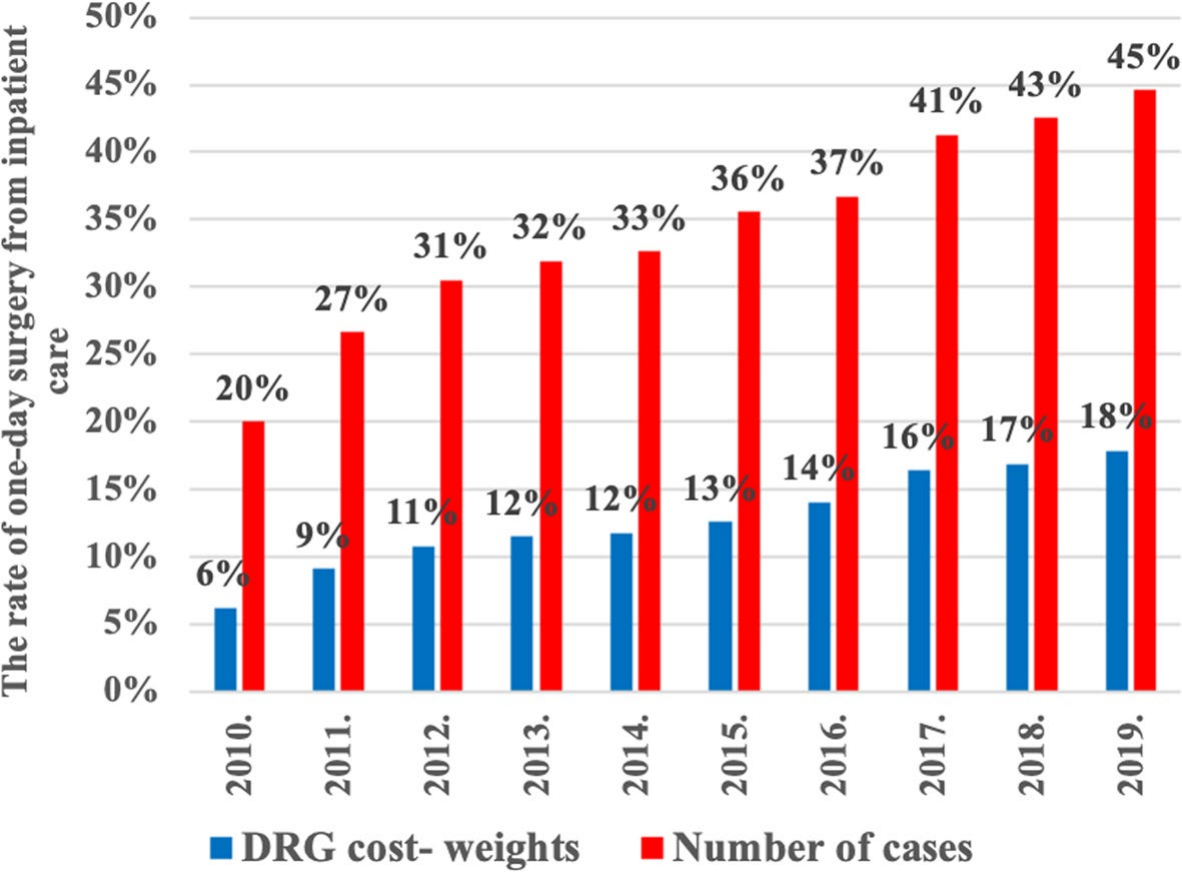
The proportion of financed one-day surgery cases and DRG cost-weights compared to the financed number of cases and DRG cost-weights of inpatient care between 2010 and 2019

Over the period examined, 2010–2019, 2,435,611 one-day surgical cases, and 1,327,280 one-day surgical DRG cost-weights were reported and reimbursed by the NHIFA in Hungary. In the study database, we did not record any mortality. We identified 28,928 cases with complications, which is 1% of the study group. Most of the complications (*n* = 16,781 or 58%) were related to obstetrics and gynecology. The sum of financed DRG cost-weights during the period investigated was 888,831,990 USD related to the one-day surgical care in Hungary. The lowest public expenditure was in 2010 (41,135,747 USD), while the highest was in 2019 (138,483,547 USD). One-day surgery cases and DRG cost-weights showed a linear increase through the years between 2010 and 2019. However, this kind of linear increase in the number of cases and DRG cost-weights of inpatient care could not be justified. Among the performance indicators of inpatient care, the scope of surgical DRGs should be highlighted: as with respect to the fundamentals of one-day surgical care, comparisons were made relating to the DRGs of inpatient care in order to clearly reveal the development of one-day surgical care in Hungary. In the first year of the research, only 20% of surgical cases belonged to one-day surgical care, however, this ratio already reached 45% in 2019. Similar increase was discovered among surgical DRG cost-weights, however, the dynamics of the increase was less remarkable as that observed in the number of cases (2010: 6%, 2019: 18%). The change in the utilization of Hungarian inpatient and one-day surgical care are shown in Table 1, and the change in the proportion of one-day surgical cases financed and DRG cost-weights compared to the inpatient care are shown in Fig. 2.

**Table 1:** The change of public expenditure related to the inpatient and one-day surgical care between 2010 and 2019

Year	Number of DRG cost-weight in inpatient care	Number of DRG cost-weight in one-day surgical care	Finance of 1 DRG cost-weight in Hungarian Forint (HUF)	Finance of 1 DRG cost-weight in US Dollar (USD)	Public expenditure for inpatient care in Hungarian Forint (HUF)	Public expenditure for one-day surgical care in Hungarian Forint (HUF)	Ratio of one-day surgical care’s expenditure compared to inpatient’s expenditure	Hungarian Forint/1 USD	Public expenditure of inpatient care in USD	Public expenditure of one-day surgical care in USD
2010.	2,221,528	57,083	150,000	721	333,229,197,767	8,562,405,716	2,57%	208.15	1,600,908,949	41,135,747
2011.	2,274,859	88,340	150,000	746	341,228,787,563	13,251,021,333	3,88%	200.94	1,698,162,574	65,945,164
2012.	2,247,074	104,481	150,000	666	337,061,033,952	15,672,118,052	4,65%	225.37	1,495,589,626	69,539,504
2013.	2,268,421	113,569	150,000	671	340,263,102,098	17,035,278,045	5,01%	223.70	1,521,068,852	76,152,338
2014.	2,304,664	118,601	150,000	645	345,699,560,838	17,790,081,323	5,15%	232.52	1,486,751,939	76,509,897
2015.	2,300,690	128,357	150,000	537	345,103,538,304	19,253,570,717	5,58%	279.46	1,234,894,219	68,895,623
2016.	2,329,430	148,503	180,000	640	419,297,367,710	26,730,504,212	6,38%	281.44	1,489,828,623	94,977,630
2017.	2,334,282	176,923	185,000	675	431,842,206,045	32,730,830,880	7,58%	274.27	1,574,514,916	119,337,991
2018.	2,346,672	188,158	198,000	733	464,641,094,442	37,255,191,774	8,02%	270.25	1,719,300,997	137,854,549
2019.	2,374,061	203,284	198,000	681	470,064,001,536	40,250,242,888	8,56%	290.65	1,617,285,400	138,483,547

**Fig. 3:**
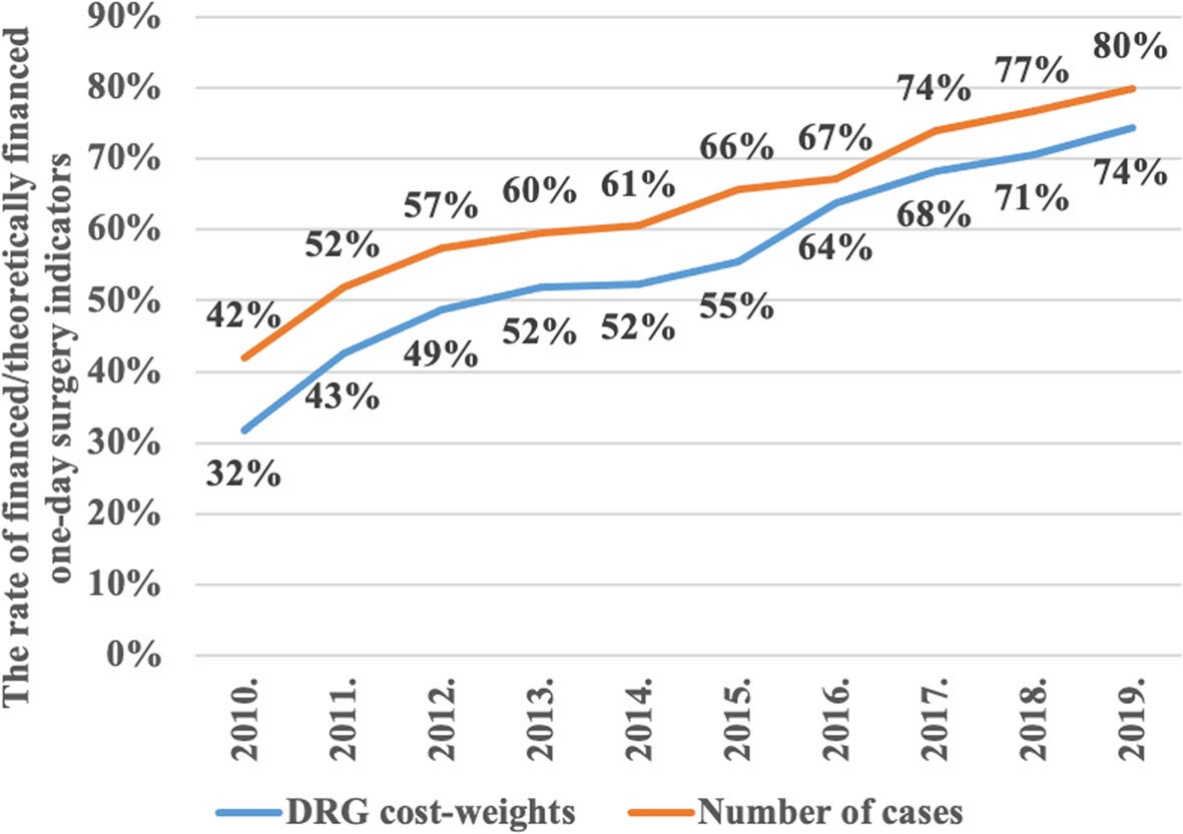
The proportion of one-day surgery cases and DRG-cost-weights compared to the theoretical maximum of number of cases and DRG cost-weights set by law in Hungary (2010–2019)

The proportion of cases and DRG cost-weights that could fall under one-day surgical care in accordance with Annex No. 9 of Public Welfare Ministerial Decree 9/1993 (IV.2.) was analyzed in the research. In 2010, only 42% of eligible one-day surgical cases were actually treated on a day surgery basis and only 32% of the maximum of related DRG cost-weights were financed by the NHFIA. In 2019, 80% of cases are eligible for one-day surgical care were actually treated in one-day surgical form by Hungarian hospitals and the related portion of the DRG cost-weights had already increased up to 74%. The years 2011, 2015, and 2017 proved to be milestones in the provision of one-day surgical care in Hungary, as policy and financing measures supporting the significant spread of one-day surgery emerged, the impact of which could already be detected. Throughout the study period, the availability and utilization of one-day surgery has grown significantly indicated by the fact that the proportion of cases and DRG cost-weights increased remarkably (Fig. 3).

**Fig. 4:**
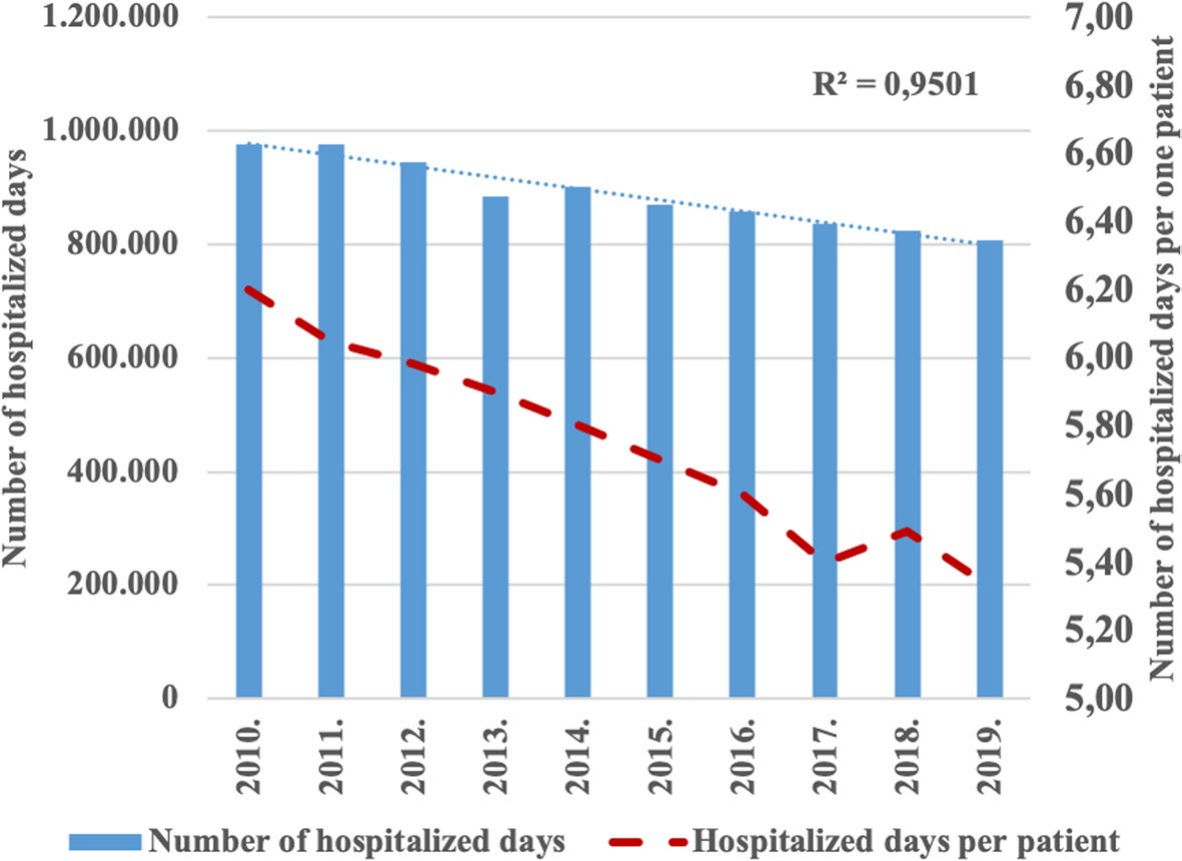
Number of hospitalized days in inpatient care during the analyzed period (2010–2019)

The continuous increase of one-day surgical care significantly affected the number of inpatient hospital days: the total number of hospital days in inpatient care was 975,746 in 2010, while in 2019 it was 806,019. The total number of hospital days in inpatient care decreased more than 160,000 days during the research period. Similar decrease was found with regard to hospital days per single patient. While the number of hospitalized days per patient in 2010 was 6.20 days, in 2019 it was 5.33 days (Fig.  4).

During the study, the value of CMI in one-day surgical care was also analyzed for each year. CMI was the lowest in 2010 (0.436) and the highest in 2019 (0.599). Between 2010 and 2019, only the year 2015 witnessed a decline in CMI (0.511). In May 2015, an important health policy initiative was introduced to the clinical and financial areas of the Hungarian health care system. As the previously applied PVL technique in one-day surgical care was abolished, it became possible for each case, which could be treated on a day-surgery basis, to be reimbursed without limitation by the NHFIA.

One-day surgery has a pivotal importance for the working-age population, as the mean age of those who were treated in one-day surgical treatment was less than 53 years old [(52.63 years, CI (95%) =5.19–86.55)]. The mean age (55.75 years) of patients who were treated in one-day surgical care in 2019 was 8.39 years higher than it was in 2010 (47.36 years). While the mean age of women who were treated in one-day surgical care between 2010 and 2019 was less than 51 years, [(50.99 years, CI (95%) = 8.25–85.98)] that of men surpassed 56 years [(56.36 years, CI (95%) =5.21–86.86)]. The mean age of women patients in the first year increased by more than 9 years during the period under investigation (2010: 44.93 years, 2019: 54.66 years); the mean age of male patients also increased, although the difference was less than 3 years (2010: 54.74 years, 2019: 57.65 years).

**Fig. 5:**
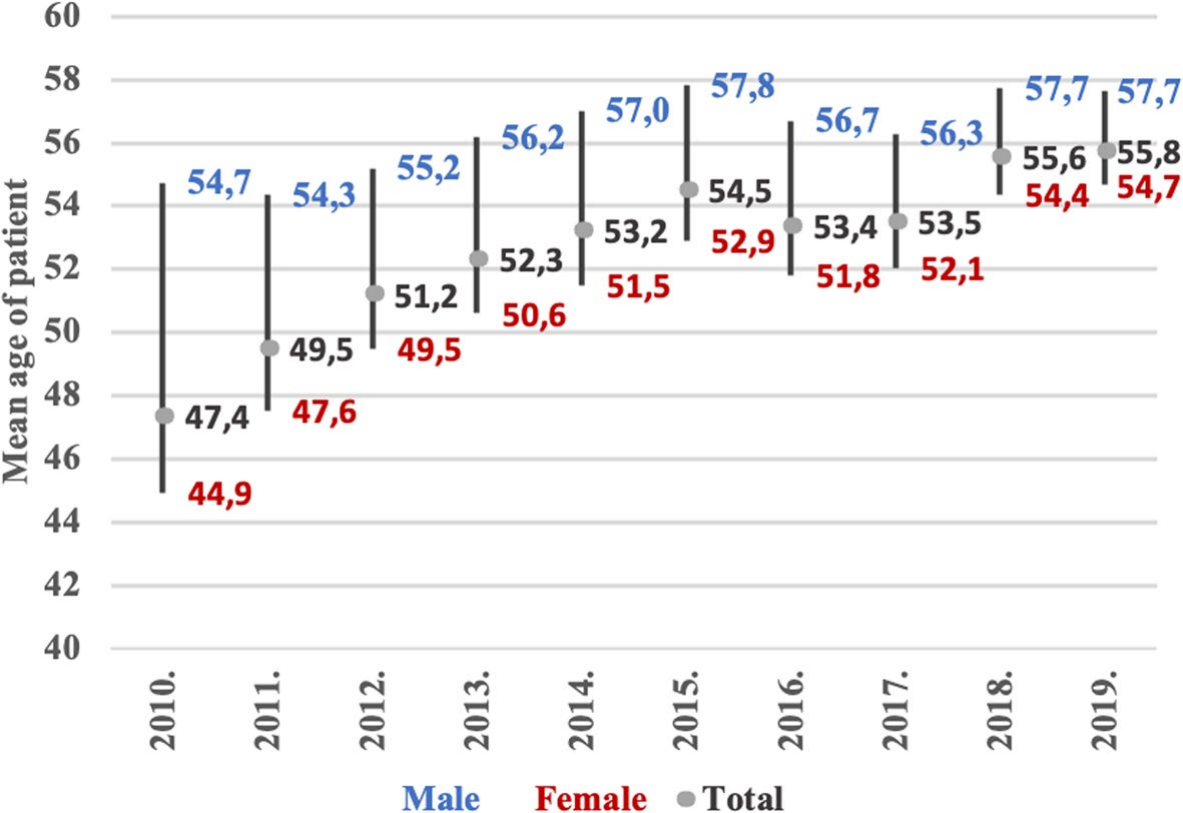
The mean age of patients undergoing one-day surgery, broken down by gender (2010–2019)

The increase of mean age in the reported cases in Hungary may be explained by the fact that the number of Hungarian citizens aged 0–14 years decreased by 53,991, and the number of citizens between15–64 years decreased by 327,515 as well, while the number of the Hungarian population over 65 years old increased by 164,743 persons (Fig. 5).

**Fig. 6:**
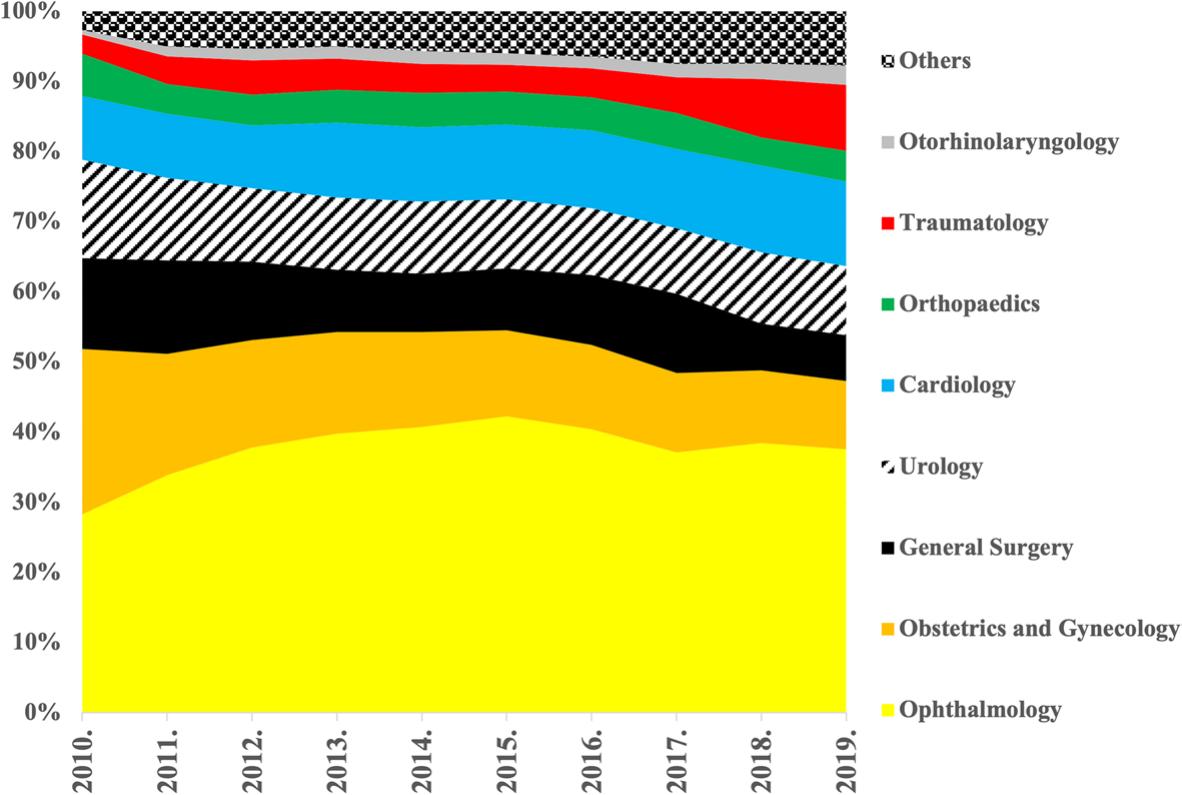
Market share of different medical professions according to financed one-day surgery DRG cost-weights (2010–2019)

The market share of one-day surgical performance was analyzed according to type of medical profession on the basis of financed DRG cost-weights (1,327,280). Most financed one-day surgery DRG cost-weights were linked to ophthalmology care (507,986 weights), which accounted for nearly 40% of the total, financed, one-day surgical DRG cost-weights between 2010 and 2019. Financed DRG cost-weights in obstetrics and gynecology –that had the second highest DRG cost-weights in the ranking – were significantly lower than that of the ophthalmology (168,939 weights). One-day surgical performance rates related to cardiology, surgery and urology were respectively similar according to market share (cardiology: 11%; surgery: 9%, urology: 10%). Those medical professions involved into the treatment of the diseases affecting the musculoskeletal system (orthopedics and traumatology) represented a similar market share in terms of financed DRG cost-weights (orthopedics: 5%; traumatology: 6%). Although, in Hungary, there are several medical interventions that are carried out in a day- surgery setting in otorhinolaryngology, the related market share was only 2%. The cumulative one-day surgical DRG-cost weights during the analyzed period (1,243,002) of the afore-mentioned 8 professions listed in Fig. 6 amounted to 94% of the whole performance financed in Hungary within one-day surgical care (1,327,280 weights).

## Discussion

The purpose of our study was to provide a comprehensive analysis of the development of Hungarian one-day surgical care in a DRG-based performance financing system, as several policy measures facilitating the broader application were implemented in Hungary in the past decade. We found that between 2010 and 2019, the market share of one-day surgical cases increased from 42, to 80%. Simultaneously the constant increase of one-day surgical cases, the number of hospitalized days were decreased in inpatient care by 17%. The value of Case Mix Index has also increased, approximately by 140%, which could confirm that more complex interventions are being conducted in one-day surgical care.

The increased utilization of one-day surgical care is a generally witnessed process throughout Europe, together with the development of this treatment modality as part of several health-related policy strategies, due to its effectiveness in a wide array of areas [8, 23]. Several publications have highlighted the advantages of one-day surgical and outpatient care, as they can effectively reduce the high costs of inpatient care which is a major challenge for health care- related policies today [24–26]. During the observed period, more than 15 billion USD were financed for Hungarian inpatient care, meanwhile the public expenditure of one-day surgery did not exceed 900 million USD. Regarding inpatient care, the annual public expenditure in 2019 was only 1% higher than it was in 2010 (the difference is: + 16 million USD), while the annual publicly financed health care expenditure related to the one-day surgery was more than 337% higher (+ 97 million USD) higher than it was in 2010.

Internationally, health policy makers follow a diverse set of practices to support the spread of one-day surgical care, however the academic literature emphasizes that besides meeting medical standards, the application of supportive funding techniques are also a major key for success [27].

There is a well-known Western European approach that identifies “innovative financing techniques” among university clinics [28] thereby, providing financial incentives for wider dissemination of interventions. A similar policy goal may have been in the background of the introduction of the 110% DRG funding in one-day surgical care in Hungary. In addition to policy support, the development of appropriate institutional DRG-coding mechanisms resulting in institution-specific optimization of DRG-based accounting for funding may be of paramount importance [29]. Previous studies have highlighted the positive association between health insurance systems and the utilization of different types of the treatment [30]. Given that the Hungarian health insurance system provides coverage for all Hungarian citizens, the use of one-day surgical care mainly depends on the medical condition of the patients and the decision of the management of the hospitals as well. Based on the Hungarian health policy initiatives, we expected and did detect a shift in everyday practice from inpatient care towards one-day surgery.

Our results showed that based on inpatient DRGs, the market share of one-day surgery increased year by year, while more and more of the interventions defined by law – that are allowed to be executed on a one-day surgery basis– are performed by Hungarian healthcare institutes. Recent years have seen several reports on the low rate of Hungarian one-day surgeries in the case of the most common intervention, cataract surgery (2007–2011: > 40%) [31]. As a result of the health policy efforts of the previous decade, − taking into account all medical professions – in less than10 years, the utilization rates of Hungarian one-day surgical care already approached the level of northern European countries [13, 32]. The country’s health policy intentions for one-day surgical care and the associated development dynamics could be well illustrated by the fact that the more economically advanced country, France, set a target of 66% market share of one-day surgical care by 2020 [33]. This has already been achieved in Hungary in 2016. There are not currently available validated one-day surgical rates published in the international academic literature that would cover all areas of the medical professions from the neighbor countries of Hungary, but several publications address recommendations for the demand to expand one-day surgical care [16, 34]. Similar to inpatient care, CMI is also an informative indicator in the performance evaluation of one-day surgery, which increased between 2010 and 2019 in Hungary. This could be explained by the significant role of university clinical centers in one-day surgical care which resulted from favorable financing techniques that were introduced– 110% basic DRG fee and PVL-free reckoning. As a result of the constantly increasing health care costs and the rapid development of surgical and anesthesiologic techniques, more and more complex diseases could be found to have contributed to this trend of increase of CMI [35] One-day surgery patients are predominantly from the working-age population, which has also been described by several previously published studies [26, 36]. It is important to highlight that even in highly costly medical professions such as interventional cardiology, procedures could be identified that are particularly costly to hospitalize. However, in a one-day surgical care, the cost of treatment per case could be significantly reduced, saving considerable resources even at the institutional level.

Considering types of medical profession or the interventions performed, the results of our study are in line with the international trend, as the importance of obstetric-gynecological, ophthalmological, cardiological, orthopedic-traumatological, general surgical, and urological interventions performed in a day- surgery setting may have multiple advantages and benefits. ^37^

## Conclusion

In the beginning of the last decade, the market share of one-day surgical care was marginal in Hungary, due to the fact that most of the less complex invasive interventions were still executed in an inpatient setting. Therefore, the amendment of health care financing legislations – mainly the abolition of volume caps and the implementation of increased DRG fee –aimed to support the widespread availability of less cost-demanding treatment modalities, consequently, there was a simultaneous increase in the number of interventions which could be concluded in one-day surgical care. The modified financing techniques contributed to the creation of a supportive environment for the spread of one-day surgical care for all public-funded Hungarian hospitals.

The institutional implementation of the afore-mentioned health policy initiatives was rather different during the period analyzed: those hospitals that provided fewer complex interventions could benefit more rapidly from the reform compared to those that produced higher CMI.

In the last few years, the supportive financing environment with regard to one-day surgical care has become an essential feature of the Hungarian health policy system, which has apparently, led to a significant increase in the market share of one-day surgical care. The increased impact of one-day surgery upon the Hungarian health care system is not only a result of advantageous financing techniques. On the one hand, potential interventions which are eligible to be executed in one-day surgical care have increased, on the other hand one-day surgical care has become less cost-demanding and provides just as equal patient safety as inpatient care. All the above may have increased trust among patients towards this form of treatment.

Given that the Hungarian reforms have achieved significant progress with regard to the proliferation of one-day surgical care, these rules and regulation could even be considered as an international best practice for those low- and middle-income countries which currently underutilize one-day surgical care.

### Limitations

The study has several limitations. Firstly, our research database did not include information on one-day surgical care provided by private health care facilities. This data is not available from publicly in Hungary. Given the lack of data, it was not possible to analyze the utilization and expenditures related to private health care, which may be of considerable interest in the international audience. If we had data from utilization of one-day surgical treatment in private health care, we would have assumed that number of cases would have increased significantly. The primary reason for this finding is that private health care institutions place a high priority on cost-effective care management, which could be already provided by one-day surgical treatment. Information from these areas would have allowed us to analyze in more depth of the effectiveness of one-day surgical care in Hungary. Secondly, the study database did not contain information about the out-of-pocket expenditures of patients who received public- funded one-day surgical treatment. Considering that the place of rehabilitation after one-day surgical procedure is transited into the patient’s home, the questions raised in this context would also have provided valuable information for the experts who are interested in the Hungarian health care system. In order to better assess the cost-effectiveness of one-day surgical treatment it is important to expand the data collection practices with the out-of-pocket expenditures of the patients in the near future in Hungary. Despite the limitation of the research assessment of the publicly financed expenditure as fully as possible from the database was aimed with the purpose of providing an international context for the incentives that, in addition to policy decisions, are also tried to facilitate the increase of market share of one-day surgical treatment by providing additional resources. Thirdly, no readmissions were identified due to complications in the study, but our database did not contain information on the whole patient pathway in one-day surgical care. For the future it has prime importance to expand the scope of the data collection in this regard in order to the effectiveness of one-day surgical treatment could be analyzed in a wider perspective. Lastly, further limitation was due to our database did not contain any information about the operating time. On the one hand, the constant development in surgical techniques could reduce the surgical stress, and support the quicker recovery of patients, on the other hand surgical procedures may also take less time due to the accelerated patient management practices in one-day surgical treatment. It would have raised the quality of the analysis if operating times according to different types of treatment had been available, because optimizing the utilization of the operating theatre is an important issue in effective care management.

## Data Availability

The data that support the findings of this study are available from National Health Insurance Fund Administrations of Hungary, but restrictions apply to the availability of these data, which were used under license for the current study, and so are not publicly available. Data are however available from the authors upon reasonable request and with permission of National Health Insurance Fund Administrations of Hungary.

## Abbreviations

WHO: World Health Organization
IAAS: International Association for Ambulatory Surgery
DRG: Diagnosis-Related Groups
PVL: Performance Volume Limit
NHIFA: National Health Insurance Fund Administration
CMI: Case Mix Index
ICPM: International Classification of Procedures in Medicine
